# Effect of acupuncture combined with rehabilitation training on sensory impairment of patients with stroke: a network meta-analysis

**DOI:** 10.1186/s12906-024-04401-9

**Published:** 2024-02-26

**Authors:** Jiaqi Wang, Bangqi Wu, Yuanyuan Tong, Xuhui Wang, Zhaojun Lu, Wenqing Wang

**Affiliations:** 1 First Teaching Hospital of Tianjin University of Traditional Chinese Medicine, Tianjin, China; 2 National Clinical Research Center for Chinese Medicine Acupuncture and Moxibustion, Tianjin, China

**Keywords:** Stroke, Acupuncture, Rehabilitation training, Sensory impairment, Network meta-analysis

## Abstract

**Background:**

The refractory and disabling nature of sensory disorders after stroke seriously affects patients' daily lives and reduces hospital turnover. Acupuncture, as an alternative therapy, is commonly used in combination with rehabilitation training to improve sensory disorders. To compare the effects of different acupuncture-related treatments combined with rehabilitation training on sensory impairment and the daily living ability of patients with stroke, we conducted a network meta-analysis to provide evidence-based findings for clinical practice.

**Methods:**

Randomized controlled trials on the treatment of sensory disorders in patients with stroke were systematically retrieved from several databases, including China National Knowledge Infrastructure (CNKI), China Science and Technology Journal(VIP), Wanfang Database, Chinese Biological Medical (CBM), PubMed, Embase, Web of Science, Cochrane Library, and Clinical trials. The retrieval period ranged from January 2012 to December 2023. Two independent reviewers screened the included literature, extracted the data, and assessed the risk quality using Cochrane Handbook 5.1.0 and ReviewManager 5.4.1. Stata16.0 software was employed for data analysis. The study protocol was registered in PROSPERO: CRD42023389180.

**Results:**

After screening, 20 studies were included, involving a total of 1999 subjects. The network meta-analysis results indicate that, compared to standard rehabilitation, acupuncture plus massage plus rehabilitation showed the most significant reduction in Numbness Syndrome Scores (MD = -0.71(-1.11,-0.31)). Acupuncture combined with rehabilitation demonstrated the most substantial improvement in Sensory Impairment Scores (MD = -0.59,(-0.68,-0.51)) and daily living ability of patients (MD = 17.16,(12.20,22.12)).

**Conclusions:**

In comparison to standard rehabilitation, the combination of acupuncture-related treatments and modern rehabilitation training not only improves the symptoms of sensory impairment and numbness after stroke but also enhances the daily living ability of patients, especially when acupuncture is combined with rehabilitation. However, further demonstration is required to strengthen these conclusions.

## Background

Stroke is a cerebrovascular disease with a high incidence rate and mortality [1]. According to the Global, Regional, and Country-Specific Lifetime Risks of Stroke in 1990 and 2016, the global average lifetime risk of stroke is increasing,with China having the highest risk at 39.3% [37.5 – 41.1] [2]. After a stroke, patients often experience various sequelae, and approximately 50% of them develop sensory disorders, particularly in the upper limbs [3]. These sensory disorders may involve tactile abnormalities, pain, vibration sensation, and proprioception loss. Clinically, patients may exhibit diminished acupuncture sensation, impaired sense of position and movement and body parts, among other symptoms [4, 5]. Proprioception, in particular, is more susceptible to damaged than the sense of touch, and the severity of sensory impairment is closely related to the stroke’s severity [5]. Furthermore, sensory disorders can significantly impact the recovery of motor function, mood and daily living abilities of patients with stroke [6, 7]. Additionally, sensory loss can prolong hospital stays and reduce discharge rates. Unfortunately, the recovery of sensory function is often overlooked, with greater emphasis placed on motor function recovery [8].

Various methods are currently employed to treat sensory disorders after stroke, including rehabilitation training, Western medicine treatments, and traditional Chinese medicine therapies. Traditional Chinese medicine therapies encompass Chinese herbal medicine, various types of acupuncture, cupping, and moxibustion. Among these, acupuncture has shown greater effectiveness in treating sensory disorders. Different types of acupuncture methods, such as body acupuncture, fire acupuncture, warm acupuncture, blood-letting puncture, electro-acupuncture, and acupoint injection have been utilized. Rehabilitation training for sensory disorders refers to any sensory training applied to the limbs or trunk to restore sensory function. Clinicians perform repeated beneficial sensory stimulation on the patient's limbs, or use instruments for precise and systematic rehabilitation to increase sensory input [8]. Repeated sensory stimulation can enhance the plasticity of the motor cortex, thereby promoting the recovery of motor function [9]. However, single treatment approaches often have slow and protracted outcomes. Therefore, although the research hotspot of combining traditional therapy with modern rehabilitation technology to treat sensory disorders after stroke is constantly heating up [10, 11], it was lacked systematic evaluation of this type of therapy. In light of this, the present study aims to conduct a network meta-analysis of literature pertaining to the treatment of post-stroke sensory disorders. The objective is to explore the combined use of acupuncture-related treatments and modern rehabilitation training, seeking to leverage the strengths of both traditional Chinese medicine and Western medicine. The findings of this study are intended to provide evidence-based guidance for the clinical management of sensory disorders following stroke.

## Methods

According to the guidelines for network meta-analysis, namely the Preferred Reporting Items for Systematic Reviews and Meta-Analyses for Network Meta-Analyses (PRISMA-NMA), We conducted the present meta-analysis, which has been registered on PROSPERO under the registration number CRD42023389180.

### Inclusion and exclusion criteria

#### Study design

We included randomized controlled trials that examined the effects of acupuncture-related treatments combined with rehabilitation training in the treatment of sensory disturbance and numbness after stroke.

### Inclusion criteria

#### Type of participants

The diagnostic criteria for stroke followed the guidelines issued by the Chinese Society of Neurology, which encompassed the Diagnostic Criteria for Cerebral Vascular Diseases, Chinese Guidelines for the Diagnosis and Treatment of Acute Ischemic Stroke 2010 [12], Guidelines for Diagnosis and Treatment of Acute Ischemic Stroke in China 2014 [13], Classification of Cerebral Vascular Diseases in China 2015 [14], Chinese Guidelines for Diagnosis and Treatment of Acute Ischemic Stroke 2018 [15], and Diagnostic Criteria of Cerebrovascular Diseases in China 2019 [16].

Sensory disorders were defined as abnormal manifestations perceived by the patient's supervisor, including shallow sensation and proprioception disorders. Main clinical manifestations comprised hypoesthesia or loss, sensory abnormality or allergy, sensory inversion or visceral discomfort. Additionally, the latency of SEP (somatosensory evoked potentials) potentials was prolonged, and the amplitude was reduced [17].

#### Type of interventions

The treatment group mainly adopts acupuncture combined with rehabilitation training. It can be combined with other traditional therapies.

The types of acupuncture include acupuncture (body acupuncture), warm needing, fire needing, electro-acupuncture, acupoint injection, and blood-letting puncture.

#### Type of comparisons

The control group underwent routine rehabilitation training.

According to the mechanism of action, rehabilitation training of sensory impairment is divided into active training and passive training [18].

Active training: Using learning principles and training methods that enhance sensory input (such as proprioception, tactile recognition, and localization).

Passive training: A sensory stimulation method that initiates the nervous system through external sensory stimuli (peripheral nerve stimulation, transcutaneous electrical nerve stimulation).

#### Type of outcome indicators

The outcome indicators included the Modified Barthel Index Score (MBI), the Scores of Numbness Symptom, and the Sensory Disturbance Scores.

1. The Modified Barthel Index Score（MBI）assessed living ability

The score contains 10 basic life items, with a total of 100 points. The higher the score, the better the patient's limb function [19].

(2) The Scores of Numbness Symptom and the Sensory Disturbance Scores.

No symptom: 0 point; Occasional symptoms: 1 point; Frequent symptoms: 2 points; Continuous delivery: 3 points. The higher the score, the more serious the symptom [20].

### Exclusion criteria

- Not related to sensation disorders after stroke.
- Control group adopted other treatments.
- Unmatched outcome indicators.
- Reviews, theoretical discussions, case reports, animal experiments, crossover trials, and non-randomized controlled trials.
- Duplicate publications and studies with incomplete data.

### Search strategy

Randomized controlled trials on the treatment of sensory disorders in patients with stroke were systematically retrieved from multiple databases, including China National Knowledge Infrastructure (CNKI), China Science and Technology Journal(VIP), Wanfang Database, Chinese Biological Medical (CBM), PubMed, Embase, Web of Science, Cochrane Library, and Clinical trials. The retrieval time was from January 2012 to December 2023. References included in the retrieved studies were also examined. Chinese and English search terms included “acupuncture, warm needing, fire needing, electro-acupuncture, acupoint injection, blood-letting puncture; cerebrovascular accident, cerebrovascular apoplexy, stroke; sensation disorders, hypesthesia”.

### Study selection and data extraction

Two reviewers independently screened the included literature using NoteExpress 3.7.0. Duplicate literature was removed, followed by reading the titles and abstracts according to the inclusion and exclusion criteria. The selected literature that met the criteria underwent full-text reading to determine inclusion in the analysis. Collect data in Excel, including first author, year of publication, country of publication, sample size (intervention group and control group), age, intervention measures (intervention group and control group), course of treatment, and outcome indicators.

### Bias risk assessment

The quality of the included study was evaluated using the bias risk assessment tool recommended in Cochrane 5.1 Handbook [21], which covered 7 aspects: random sequence generation; allocation concealment, blinding of subjects and researchers, blinding of outcome assessment, completeness of outcome data, selective reporting, and other biases. The risk of each item was judged sequentially, and the results were visualized using ReviewManager 5.4.1 software. The two reviewers exchanged and compared their evaluation results, with any differences resolved through collective discussion by the research group.

### Statistical methods

We used Stata 15.0 and ReviewManager 5.4.1 to conduct statistic analysis. For dichotomous outcome, we used odds ratio(OR) and 95% confidence interval (CI) to quantify the effectiveness. For continuous outcome evaluated using the same scale, the weighted mean difference (WMD) and 95% confidence interval (CI) were employed as the effect size. Given the clinical and methodological heterogeneity of the selected methods and subjects in the trials, a random-effects model was chosen for statistical analysis [22]. Network funnel plots and Egger's test were performed to assess publication bias [23, 24]. The surface under the cumulative ranking curve (SUCRA) was used to rank the advantages and disadvantages of interventions.

## Results

### Study selection and study characteristics

**Fig. 1:**
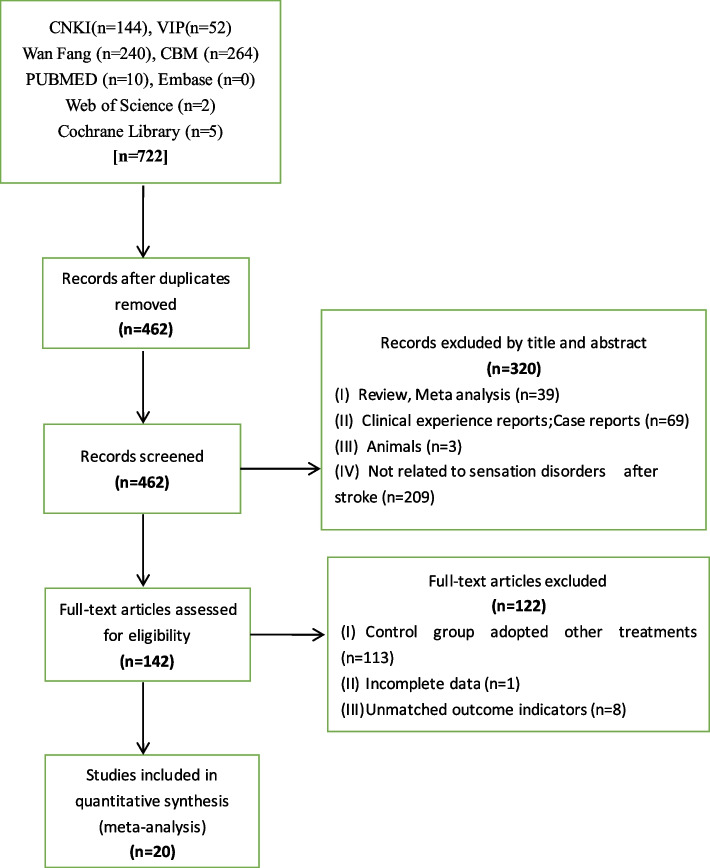
PRISMA flow diagram

Following preliminary screening based on the title and abstract, 142 articles were obtained, and their full texts were read. After careful examination, 122 articles were excluded due to intervention measures, diagnostic criteria, and non-randomized controlled experiments. Finally, 20 articles were included in the analysis [11, 25–43] (Fig. 1).

The total sample size was 1999 cases, including 1001 cases in the treatment group and 998 cases in the control group. The study involved 11 interventions, including acupuncture plus rehabilitation, acupuncture plus massage plus rehabilitation, acupuncture plus oral Chinese medicine plus rehabilitation, warm needing plus rehabilitation, acupoint injection plus oral Chinese medicine plus rehabilitation, acupoint injection plus rehabilitation, electro-acupuncture plus rehabilitation, acupuncture plus herbal fomentation plus rehabilitation, blood-letting puncture plus rehabilitation and rehabilitation. Tables 1 and 2 provide details of the characteristics of the included studies.

**Table 1:** Characteristics of included studies(1)

	Study	Intervention		Number of patients (T/C)	Intervention period	Relevant outcomes
		T	C			
1	Ma Huimin 2022	J	K	22(11/11)	14days	①
2	Jia Xiaonan 2022	B	K	107(54/53)	30days	②
3	Wang Wei 2022	C	K	67(34/33)	60days	②
4	Li Hongying 2022	A	K	76(38/38)	90days	②
5	Li Lingling 2021	G	K	66(33/33)	60days	①
6	Chen Yinjianzi 2021	G	K	102(51/51)	14days	①②③
7	Ye Zhong 2021	A	K	40(20/20)	30days	①
8	Li Yawei 2021	D	K	120(60/60)	30days	②
9	Lv Sunsun 2021	D	K	80(40/40)	28days	①
10	Wu Suqing 2021	B	K	440(220/220)	56days	①
11	Wu Yuping 2021	H	K	100(50/50)	14days	②
12	Bai Xiaohui 2021	A	K	78(39/39)	28days	②
13	Wang Pan 2021	D	K	90(45/45)	20days	②③
14	Hua Bin 2021	A	K	40(20/20)	42days	②
15	Yan Li 2020	A	K	156(78/78)	56days	①
16	Zhao Wenjin 2018	C	K	80(40/40)	84days	②③
17	Dong Gang 2017	F	K	89(45/44)	60days	①②③
18	Feng Xiaodong 2015	I	K	60(30/30)	28days	①
19	Wang Hongbin 2015	A	K	104(52/52)	14days	②③
20	Xiong Peifang 2015	I	K	82(41/41)	28days	①②

*Abbreviation*: *T* Treatment groupm, *C* Control group

①Modified Barthel Index (MBI) ②The Scores of Numbness Symptom.③The Sensory Disturbance Scores

A: acupuncture plus rehabilitation, B: acupuncture plus massage plus rehabilitation, C: acupuncture plus oral Chinese medicine plus rehabilitation, D: warm needing plus rehabilitation, E: acupoint injection plus oral Chinese medicine plus rehabilitation, F: acupoint injection plus rehabilitation, G: electro-acupuncture plus rehabilitation, H: acupuncture plus herbal fomentation plus rehabilitation, I: blood-letting puncture plus rehabilitation, J: fire needing plus rehabilitation, K: rehabilitation

**Table 2:** Characteristics of included studies(2)

Study	Year	Study period	Age (years) (mean ± SD)	
			T	C
Ma Huimin	2022	2020.9—2021.3	66.45 ± 8.98	64.55 ± 6.89
Jia Xiaonan	2022	2019.8–2020.12	58.63 ± 4.05	57.25 ± 4.05
Wang Wei	2022	2018.1—2019.1	56.24 ± 1.05	56.81 ± 1.16
Li Hongying	2022	2018.2–2020.7	68.81 ± 8.92	68.75 ± 8.97
Li Lingling	2021	2018.3—2019.3	58.6 ± 3.5	58.9 ± 3.1
Chen Yinjianzi	2021	2018.11–2019.11	60.21 ± 8.57	60.15 ± 8.42
Ye Zhong	2021	2018.7–2019.12	57. 2 ± 4.3	56. 3 ± 4.6
Li Yawei	2021	2019.6—2020.6	55.38 ± 11.05	56.02 ± 11.23
Lv Sunsun	2021	2018.6 ± 2019.12	58.85 ± 5.36	58.79 ± 5.28
Wu Suqing	2021	2018.1 —2020.1	57. 4 ± 5. 2	58. 9 ± 7. 4
Wu Yuping	2021	2018.1–2020.6	55.39 ± 3.41	55.36 ± 3.21
Bai Xiaohui	2021	2018.9–2019.11	60.24 ± 3.58	61.32 ± 3.62
Wang Pan	2021	2017.5–2019.10	60. 85 ± 7.64	60. 82 ± 7. 61
Hua Bin	2021	2018.2–2020.2	63.01 ± 6.08	62.15 ± 7.25
Yan Li	2020	2015.6—2018.6	62.98 ± 8.40	63.749 ± 9.91
Zhao Wenjin	2018	2015.2–2017.1	61.52 ± 8.37	60.23 ± 8.29
Dong Gang	2017	2015.1–2017.1	72.4 ± 4.6	73.6 ± 4.8
Feng Xiaodong	2015	2013.2–2014.8	56.73 ± 9.32	53.27 ± 11.62
Wang Hongbin	2015	2013.8–2014.10	56.7 ± 2.5	54.2 ± 3.6
Xiong Peifang	2015	2012.3–2013.12	65.00 ± 2.00	65.00 ± 2.00

*Abbreviation*: *SD* Standard deviation, *T* Treatment group, *C* Control group

### Bias risk assessment

**Fig. 2:**
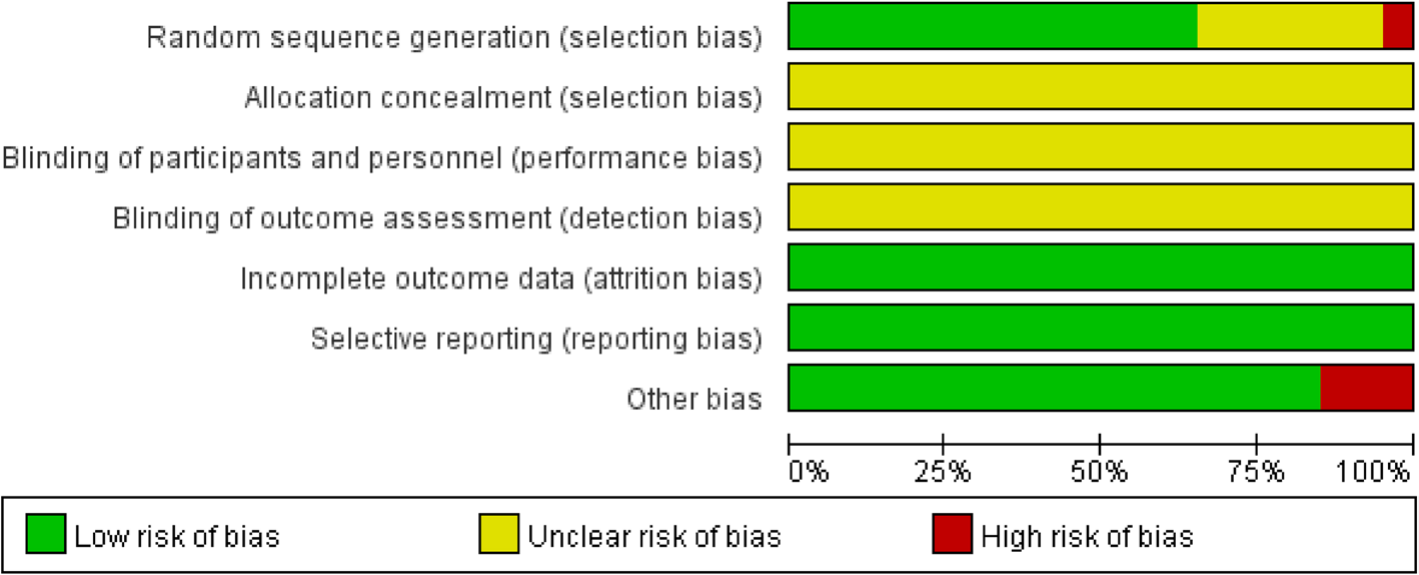
Risk of bias graph

**Fig. 3:**
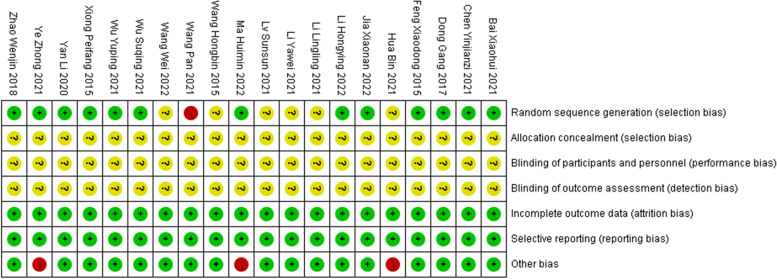
Risk of bias summary. Note: Green: low risk of bias; Yellow: some concerns; Red: high risk of bias

All 20 selected studies were randomized controlled trials [11, 25–43]. Regarding the random allocation method, 1 article was rated as high risk due to allocated based on the treatment method [37]. The 14 articles were rated as low risk as they were assigned in sequence using the random number table method [11, 25–28, 30, 31, 35, 37, 39–43]. The other 6 articles did not specify the specific allocation method [29, 32–34, 36, 38]. In terms of allocation concealment, blinding and measurement bias, all 20 articles were not clearly described. However, in terms of follow-up bias, all the literature provided clear explanations of the completeness of each indicator and the reasons for any missing or excluded data, resulting in a low risk rating. Regarding reporting bias, all 20 articles were rated as low risk. 3 articles with small sample sizes (less than 30 in each group) were rated as high risk [11, 29, 35]. No included literature mentioned other factors that could cause bias risks, so all studies were rated as unknown risk in terms of other biases (Figs. 2 and 3).

### Network meta-analysis of results

**Fig. 4:**
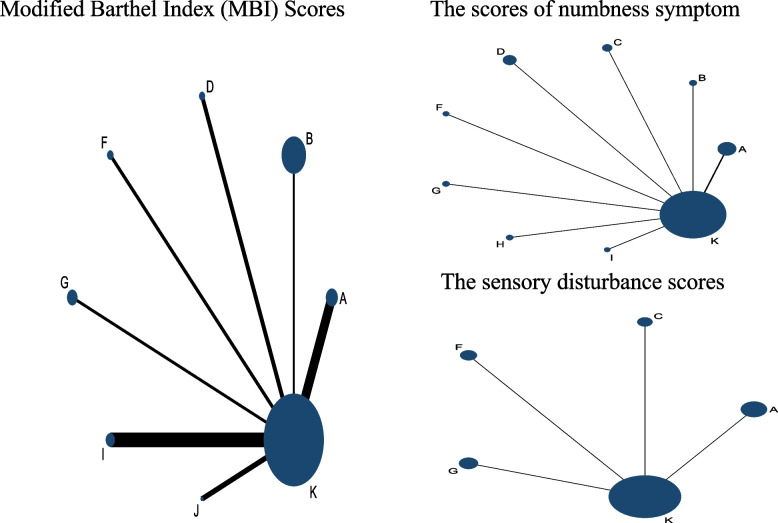
Network plot of treatment comparisons in the network meta-analysis

Figure 4 presents the network meta-analysis diagram of the effects of different types of therapeutic interventions on the Scores of Numbness Symptom, the Sensory Disturbance Scores, the Modified Barthel Index (MBI). The line between the two circles indicated there is a direct comparison between the two interventions. The size of the circle represents the sample size included in each intervention, and the thickness of the line represents the number of studies included between the two interventions.

**Fig. 5:**
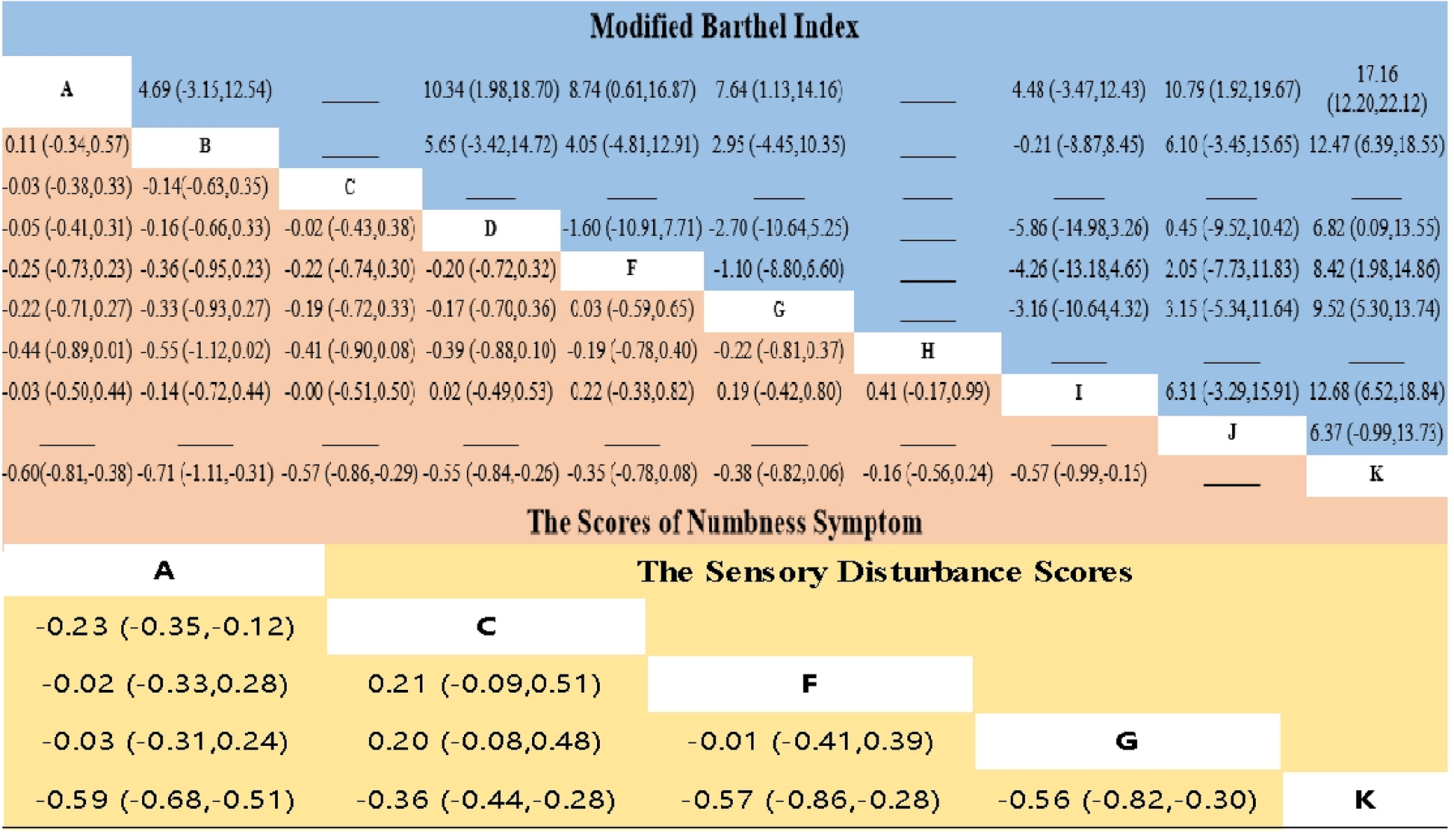
Network meta-analysis of results

Figure 5 details the complete matrix of results.

**Fig. 6:**
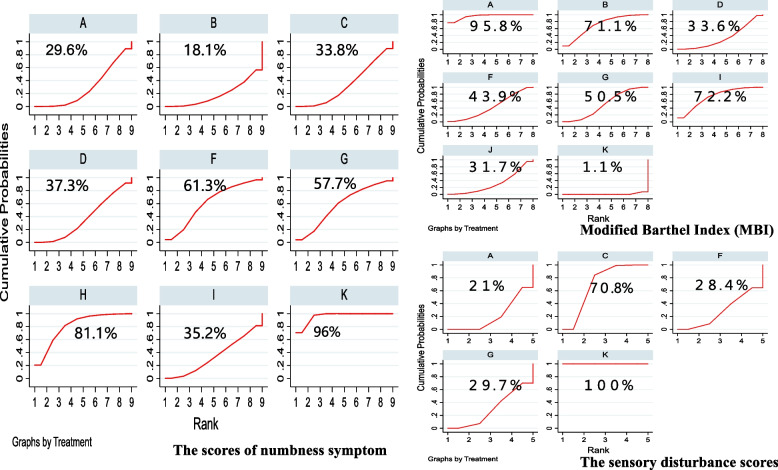
Cumulative probabilities plots of the surface under the cumulative ranking curves (SUCRAs). [Abbreviation: **A** Acupuncture plus rehabilitation, **B** Acupuncture plus massage plus rehabilitation, **C** Acupuncture plus oral Chinese medicine plus rehabilitation, **D** Warm needing plus rehabilitation, **E** Acupoint injection plus oral Chinese medicine plus rehabilitation, **F** Acupoint injection plus rehabilitation, **G** Electro-acupuncture plus rehabilitation, **H** Acupuncture plus herbal fomentation plus rehabilitation, **I** Blood-letting puncture plus rehabilitation, **J** Fire needing plus rehabilitation, **K** Rehabilitation]

Figure 6 displays the results of the surface under the cumulative ranking curve (SUCRA), which ranks all treatment plans. In the MBI scores, the higher the score of SUCRA, the more effective the method is in improving the sensory function and activities of daily living of patients with stroke. The scores of numbness symptom and the sensory disturbance scores belong to the evaluation indicators. The smaller the score, the better the efficacy.

#### Modofied barthel index

Of the 20 articles included articles, 10 reported MBI [11, 26–28, 32, 34, 35, 39, 41, 42]. Compared to standard rehabilitation, the interventions that were found to have the most significant effect on improving patients' daily living ability were acupuncture plus rehabilitation [MD = 17.16, 95%CI(12.20,22.12)], blood-letting puncture plus rehabilitation [MD = 12.68, 95%CI(6.52,18.84)], and acupuncture plus massage plus rehabilitation [MD = 12.47, 95%CI(6.39,18.55)]. According to the SUCRA results, the top three interventions based on MBI scores were acupuncture plus rehabilitation (95.8%), blood-letting puncture plus rehabilitation (72.2%), and acupuncture plus massage plus rehabilitation (71.1%).

#### The scores of numbness symptom

13 studies reported a change in the scores of numbness symptom after treatment [25–27, 29–31, 33, 36–38, 40, 41, 43]. Interventions that significantly reduced the scores of numbness symptom compared to standard rehabilitation included acupuncture plus massage plus rehabilitation [MD = -0.71, 95%CI(-1.11,-0.31)], acupuncture plus rehabilitation [MD = -0.60, 95%CI(-0.81,-0.38)], acupuncture plus oral Chinese medicine plus rehabilitation [MD = -0.57, 95%CI(-0.86,-0.29)], and warm needing plus rehabilitation [MD = -0.55, 95%CI(-0.84,-0.26)]. The difference were statistically significant (*P* < 0.00001).

The numbness syndrome score is an evaluation index, and the smaller the SUCRA value, the more effective it is. According to the SUCRA results, acupuncture plus massage plus rehabilitation(18.1%), acupuncture plus rehabilitation(29.6%), and acupuncture plus oral Chinese medicine plus rehabilitation(33.8%) were found to be highly effective in reducing numbness in patients after stroke.

#### The sensory disturbance scores

Five articles reported the syndrome score of sensory impairment [26, 27, 29, 36, 43]. Interventions that significantly reduced the sensory disturbance scores compared to the control group included acupuncture plus rehabilitation [MD = -0.59, 95%CI(-0.68,-0.51)], acupoint injection plus rehabilitation [MD = -0.57, 95%CI(-0.86,-0.28)], electro-acupuncture plus rehabilitation [MD = -0.56, 95%CI(-0.82,-0.30)], and acupuncture plus oral Chinese medicine plus rehabilitation [MD = -0.36, 95%CI(-0.44,-0.28)]. The most effective intervention in reducing sensory disorders was found to be acupuncture plus rehabilitation (SUCRA = 21%).

### Risk of bias across the studies

**Fig. 7:**
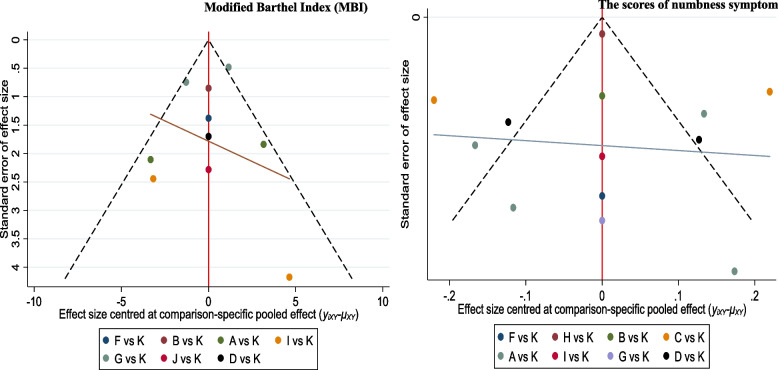
Adjusted network funnel plot for assessing publication bias in the network meta-analysis

Figure 7 presents the funnel plots of each outcome indicator. The funnel plot of the sensory disturbance scores (*P* = 0.328 > 0.5), and the Modified Barthel Index (MBI) (*P* = 0.277 > 0.5) are almost symmetrical, indicating that research publication bias is small. However, the funnel chart of the scores of numbness symptom is not completely symmetrical, and some points fall on the bottom and outside of the funnel chart, suggesting a certain degree of publication bias (*P* = 0.03 < 0.5). Therefore, interpretation of the research results should be treated with caution.

### The GRADE grading results

The quality of evidence for the study outcome indicators was evaluated following the GRADE grading process in the network meta-analysis, which included 5 downgrading factors: risk of bias, imprecision, incoherence, inconsistency, and publication bias. The final quality of evidence was graded as high, moderate, low, and very low. The GRADE grading results indicated that the quality of evidence for the comparison between interventions was generally low or very low, except for the comparison of Acupuncture plus Rehabilitation or Acupuncture plus Massage plus Rehabilitation vs Rehabilitation,which was rated as moderate. The lack of mention of blinding and allocation concealment in most of the literature contributed to an overall low quality of evidence rating.

## Discussion

In many countries, stroke is a leading cause of disability, placing a significant burden on families and healthcare systems [44]. The involvement of the sensory system is crucial for our perception, reaction, and external responses. Abnormal sensations after a stroke can hinder the recovery of a patient's motor function, leading to difficulties in performing daily activities and self-care [45, 46]. Stroke rehabilitation is a complex process, but recognized guidelines for the optimal time, type, and intensity of rehabilitation are not immediately available. Thus, clinicians need to understand how different rehabilitation programs impact the central nervous system and determine the most effective rehabilitation training strategy based on each patient's individual circumstances, including the timing, type, and intensity of rehabilitation training [47].

With advancements in medicine, clinicians have come to recognize the significance of restoring sensory function recovery for overall motor function recovery [48]. As a traditional medical therapy in China, acupuncture has gradually gained recognition as one of the most widely used and effective alternative therapies [49]. Faced with a variety of treatment options, clinicians require robust evidence to make informed decisions. Therefore, this study analyzed nearly a decade's worth of randomized controlled trials, comparing various acupuncture-related methods with rehabilitation treatment and standard rehabilitation. The study involved 11 different acupuncture-related treatment approaches, and the interventions were ranked comprehensively based on the final results. Additionally, the study identified 10 treatment methods that, when combined with rehabilitation training, showed efficacy and merit further exploration.

This study represents the first network meta-analysis of acupuncture-related treatments combined with rehabilitation therapy for post-stroke sensory disorders. The mechanism of acupuncture in treating sensory disorders after stroke involves the functional reconstruction and rehabilitation of the central nervous system [50]. By comparing the results of Single Photon Emission Computed Tomography (SPECT) perfusion and functional MRI scans taken before and after treatment, researchers found that traditional acupuncture increased cerebral blood flow in the sensorimotor area and activated the somatosensory cortex in patients with stroke, providing evidence for the therapeutic effect of traditional medicine in stroke rehabilitation [51, 52]. Many studies have demonstrated that somatosensory training and physical exercises can be conducted simultaneously, significantly activating both the sensory and motor cortex [53, 54]. Therefore, the combination of traditional acupuncture and rehabilitation training may accelerate the process of sensory recovery, enhance a patient's self-care abilities, and potentially lead to shorten hospital stays.

Despite the positive findings from the network meta-analysis, several limitations should be acknowledged. First, many of the included randomized controlled trials had small sample sizes and were conducted at single centers, potentially introducing bias into the results. Secondly, all the study subjects were Chinese, so the generalizability of the research findings to patients in other countries may need to be considered. Finally, the overall quality of the included trials was moderate, indicating a need for higher-quality and more standardized randomized controlled trials in the future.

## Conclusions

The research results demonstrate that the combination of acupuncture and rehabilitation training can significantly alleviate sensory disorders such as numbness and pain in patients after a stroke, leading to improve their self-care abilities and overall quality of life. Notably, the interventions of acupuncture plus rehabilitation and acupuncture plus massage plus rehabilitation showed particularly promising outcomes. Additionally, the other 9 acupuncture-related treatments mentioned in the study also demonstrated efficacy and warrant further investigation.

## Data Availability

All data generated or analysed during this study are included in this published article.
